# Comprehensive Analysis of Thrombotic Microangiopathy Following Renal Transplantation

**DOI:** 10.1155/ijne/4396051

**Published:** 2024-12-24

**Authors:** Ittai Fattal, Tali Steinmetz, Natalie Donin, Ana Foigelman Tobar, Benaya Rozen-Zvi, Ruth Rahamimov, Eviatar Nesher, Idit Shirazi, Eytan Mor, Ilan Babai, Zvi Fishelson

**Affiliations:** ^1^Nephrology and Hypertension Unit, Rabin Medical Center—Beilinson Hospital, Petach Tikva 4941492, Israel; ^2^Faculty of Medical and Health Sciences, Tel Aviv University, Tel Aviv 6997801, Israel; ^3^Department of Cell and Developmental Biology, Faculty of Medical and Health Sciences, Tel Aviv University, Tel Aviv 6997801, Israel; ^4^Department of Pathology, Rabin Medical Center—Beilinson Hospital, Petach Tikva, Israel; ^5^Department of Transplantation, Rabin Medical Center—Beilinson Hospital, Petach Tikva 4941492, Israel; ^6^Clinical Immunology Laboratory, Rabin Medical Center—Beilinson Hospital, Petach Tikva 4941492, Israel; ^7^Transplantation Unit, Department of Surgery B, Sheba Medical Center, Tel Hashomer, Ramat Gan 52621, Israel

## Abstract

**Background:** Thrombotic microangiopathy is a severe complication of renal transplantation. Little is known about risk factors, incidence of autoantibodies against complement components, and prognosis.

**Methods:** Clinical and laboratory data were retrospectively collected for 13 patients diagnosed with post-transplant thrombotic microangiopathy (PT-TMA) in 2011–2018. Enzyme-linked immunosorbent assay (ELISA) results were compared to transplant recipients without PT-TMA and healthy controls.

**Results:** Nine patients (69%) had potential PT-TMA risk factors other than exposure to calcineurin inhibitors (CNIs). Stratification by time to PT-TMA yielded two groups. Patients diagnosed within 6 months of transplantation (*n* = 6) were characterized by positive donor-specific antibody (DSA) test, complement-associated renal disease, and acute rejection. Two had IgG and IgA autoantibodies to complement Factors H and I, respectively. Patients diagnosed ≥ 3 years after transplantation (*n* = 7) had a high rate of infection. Renal biopsy yielded dense deposits in 6 patients, and only one with primary immune complex renal disease. Within 2 years, graft failure requiring dialysis occurred in 6 patients (46%). Three patients with early-onset PT-TMA showed improved renal function and remained stable under eculizumab treatment. Epstein–Barr virus (EBV)–associated post-transplant lymphoproliferative disorder (EPTLD) developed in 3 patients, 2 of whom had received eculizumab for more than 5 years. Five patients (39%) died during follow-up.

**Conclusion:** In this study, PT-TMA was associated with other risk factors besides CNI exposure, with differences by time of onset from transplantation. Prognosis was generally poor but better for early-onset PT-TMA managed with eculizumab. The development of late EPTLD in 3 patients raises concerns.

## 1. Introduction

Thrombotic microangiopathy (TMA) is a rare and destructive complication of renal transplantation caused by dysregulated activation of the complement system. The pathogenesis is unclear and thought to be multifactorial. Calcineurin inhibitors (CNIs) are considered the major cause in about 50% of cases, and it is recommended that their use be stopped when the disease occurs [1]. Other risk factors potentially linked to post-transplant TMA (PT-TMA) are infection, pathogenic variants in complement genes, antibodies directed at complement components, primary complement–associated renal disease, donor-specific antibodies (DSAs), and acute antibody–mediated rejection [2–7]. However, their relative impact on the development of the disease is unknown.

A pathogenic interaction between autoantibodies and regulatory proteins of the complement system has been proposed as the underlying pathophysiology of PT-TMA. IgG, IgM, and IgA autoantibodies against complement components have been described in association with atypical hemolytic uremic syndrome (HUS) and are thought to enhance the deleterious action of the complement system and cause the disease [3, 8, 9]. However, evidence from other diseases shows that autoantibodies, especially IgM subtype, are not necessarily pathogenic and can even be protective [10].

Renal transplant recipients have a high incidence of malignancies due to the long-term immunosuppressive therapy [11]. It is not known whether complement deficiency or inhibition during treatment with eculizumab increases the risk of malignancy necessitating a new cancer surveillance approach.

The aim of this study was to evaluate the background data, risk factors, and prognosis of a series of renal transplant recipients who met the diagnostic criteria for PT-TMA.

## 2. Methods

### 2.1. Setting and Patients

Clinical and laboratory data were retrospectively collected for 13 patients diagnosed with PT-TMA at a tertiary university medical center in 2011–2018. Candidates for the study fulfilled all the following criteria within 30 days: (a) lactate dehydrogenase (LDH) level above 500 U/L, (b) platelet count below 130 K/*μ*L, (c) hemoglobin below 10 g/dL, (d) schistocytes above 10 per × 50 magnification, or low level of haptoglobin, or thrombi in the renal biopsy, and (e) low C3 level. Only those who underwent renal biopsy within 6 months of the PT-TMA were included. Renal biopsies were performed at the discretion of the attending physicians. Patient 2 underwent transplantation and was diagnosed with PT-TMA at another hospital and later continued follow-up at our institution. Since we could not obtain all the laboratory results, we relied on their diagnosis. (A full description of this patient's clinical course appears in Supporting Information [available *here*]).

Patients with autoimmune diseases were excluded.

Acute antibody–mediated rejection was defined according to the Banff 2022 criteria [12]. Infection-associated PT-TMA was suspected in cases of hospital admission for infection, or infection requiring surgical intervention, or positive blood culture in 3 months before the PT-TMA episode. Findings on urinary cultures taken during the 3 months before the episode were examined as well. A positive DSA test was defined as mean fluorescent intensity ≥ 4000 units at 6 months before or after the diagnosis of PT-TMA. Biopsies were reviewed by a renal pathologist. Immunofluorescence was considered positive if the result was +1 or above. Enzyme-linked immunosorbent assay (ELISA) results were compared with age- and sex-matched transplant recipients without PT-TMA and healthy subjects.

The study was approved by the local institutional Helsinki Committee.

### 2.2. ELISA

IgG, IgM, and IgA antibodies against Factor H (FH) and Factor I (FI) were tested in patients with either a low plasma C3 level or positive protein electrophoresis and immunofixation (PEP + IF) tests in the year before inclusion in the study. As data collection started in 2016 and the study included patients diagnosed from 2011 to 2018, at the time of the study onset, only 9 patients (nos. 1, 2, 3, 5, 6, 8, 10, 11, and 13) had active serology and were tested. We compared them to age- and sex-matched 10 healthy subjects and 11 renal transplant recipients without PT-TMA, which in addition were matched in their time from transplantation. Microtiter 96-well plates were coated overnight at 4°C with FH or FI (CompTech, Tyler, TX) in 0.2 M carbonate/bicarbonate buffer, 25 ng per well, washed with tris-buffered saline (TBS), and blocked with 1.5% bovine serum albumin (BSA) in TBS. Sera were diluted 1:50 in phosphate-buffered saline (PBS)-tween containing 2% BSA, and 50 *μ*L were added to each well. Following incubation at 37°C for 1 h, the wells were washed with TBS. Peroxidase-labeled anti-IgG, anti-IgM, or anti-IgA antibodies (Jackson ImmunoResearch, West Grove, Pennsylvania) were added to the wells, incubated at 37° for 1 h, and diluted according to the manufacturer's instructions. The wells were then washed with TBS. TMB one component microwell substrate (Southern Biotech, Birmingham, Alabama) was added to each well, and the absorbance (OD) was read at 450 nm. A titration curve was formulated for each study within the limits of 0.005–0.1 mg/mL. Each ELISA test was performed twice in triplicate.

### 2.3. Genetic Analysis

Genetic analysis was performed using a targeted sequencing panel that captures 13 genes implicated in the TMAs, including *CFH, CFI, MCP (CD46), CFB, CFHR5, C3, THBD, DGKE, PLG, ADAMTS13, MMACHC, G6PD*, and *WT1*, as previously described [13]. The *CFH-CFHR* genomic region was screened for copy number variation by multiplex ligation–dependent probe amplification (MLPA) using the SALSA reagent kit (MRC Holland, Amsterdam, the Netherlands) and in-house–designed probes.

### 2.4. Protein Electrophoresis and Immunofixation

PEP + IF tests for immunoglobulins were considered if they were performed 3 years before or after the PT-TMA episode. Protein electrophoresis was performed with Sebia capillary instruments (Sebia, Gaillon, France). Sera showing abnormal peaks were then analyzed by immunofixation using the Hydrasys 2 Scan (Sebia).

### 2.5. Statistical Analysis

Student's t-test was used to compare IgG, IgM, and IgA reactivities between study groups. The Chi-square test was used to compare discrete variables between PT-TMA subgroups.

## 3. Results

### 3.1. Patients

Between the years 2011 and 2018, 1906 renal transplant recipients were followed up in our nephrology unit. Of these, 20 patients (1.1%) met the laboratory criteria. Of them, one patient was excluded because she suffered from an autoimmune disease and six were excluded because they did not have a renal biopsy. Of the 13 patients that entered the study, 8 were female and 5 were male patients with a mean age of 41 years. Mean laboratory levels of the cohort were as follows: creatinine 2.5 ± 1.4 mg/dL, C3 71 ± 11.6 mg/dL, hemoglobin 8.8 ± 0.7 g/dL, platelets 91 ± 37.2 K/*μ*L, and LDH 824 ± 276 U/L. The haptoglobin level was decreased in 67% of patients, schistocytes were positive in 82%, and C4 was decreased in 15%. Only one patient (no. 5) had both normal schistocyte and haptoglobin values. Results of the direct Coombs test were available for 10 patients and were negative in all (Table 1).

### 3.2. Potential Risk Factors for PT-TMA

The prevalence of the following potential risk factors for PT-TMA was evaluated: acute antibody–mediated rejection, primary complement–associated renal disease, antibodies directed to FH or FI, pathogenic variants in complement genes, and infection.  Table 2 summarizes the findings for each patient. Nine patients had at least one additional potential risk factor besides CNI exposure. Exceptions were Patients 3, 8, 9, and 13.

### 3.3. Early and Late PT-TMA

Analysis of the lag time to PT-TMA yielded two groups of patients: onset of PT-TMA within 6 months after transplantation (early-onset group) and onset 3 years or more after transplantation (late-onset group) (Table 3).

PT-TMA occurred early in 6 patients (nos. 1–6, 46%); in 3 (50%), the primary renal disease was complement-associated renal disease (atypical HUS, dense deposit disease, and C3 glomerulonephritis). Antibody-mediated rejection occurred or was suspected in one patient each (33%). DSA was positive in 4 patients (66%), and 2 of the 5 patients tested (40%) had antibodies against FH or FI. PT-TMA occurred late in 7 patients (nos. 7–13, 54%). None had acute rejection or antibodies directed against FH or FI, and DSA was negative in all 4 patients tested. Only one had complement-associated renal disease, but only 3 patients who had suspected infection-associated PT-TMA were in this group (Tables 3 and 4). There was no statistically significant difference between the early- and late-onset groups in levels of C3, hemoglobin, platelets, LDH, haptoglobin, and baseline creatinine (Table 1).

### 3.4. PT-TMA Associated With Infections

Three patients had evidence of infection. Patient 10 was hospitalized because of a flu-like viral infection that occurred 21 days before the PT-TMA episode, Patient 11 had buccal cellulitis due to a tooth infection that required surgical intervention 66 days before the PT-TMA episode, and Patient 12 had viral meningitis 13 days before the PT-TMA episode. All 3 had undergone transplantation more than 12 years before PT-TMA occurred. In 3 other patients, urinary cultures were positive for *E. coli* or *Enterococcus* (Patients 3 and 7) or *Candida* (on repeated cultures; Patient 13) within 50 days preceding the PT-TMA episode. None had clinical manifestations of infection, and they were not treated.

### 3.5. Genetic Analysis

Pathogenic or pathogenetic variants were not identified in any complement gene in any of the patients. Four patients were heterozygous and one was homozygous for deletion of *CFHR3-CFHR1*. This deletion is a common copy number variant in the general population, with an allele frequency of 42% in Africans, 19.8% in Europeans, 17.8% in Hispanics, and 5.7% in Asians [14]. The deletion of both alleles is present in 16.2% of Africans, 4.1% of Europeans, 1.9% of Latinos, and 0.3% of East Asians. (Data based on the reported nondiploid CN frequency in gnomAD).

### 3.6. Anti-FH and Anti-FI Antibodies

**Figure 1:**
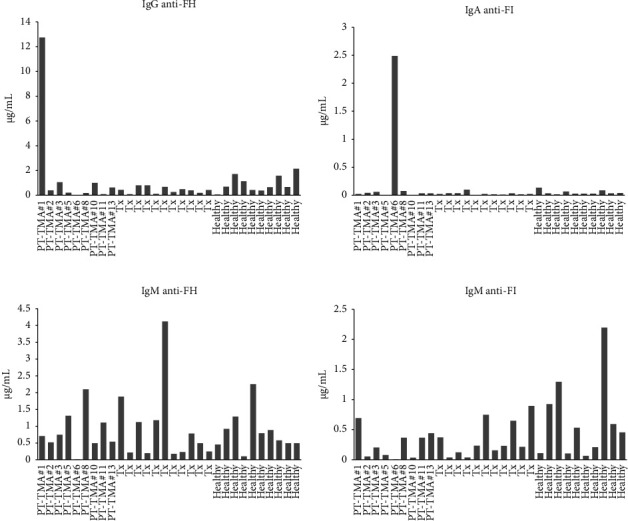
Sera from 9 transplanted patients with PT-TMA, 10 transplanted patients without PT-TMA, and 11 healthy subjects were tested by ELISA for anti-FH and -FI antibodies. (a) Detection of IgG antibodies to FH. (b) Detection of IgA antibodies to FI. (c) Detection of IgM antibodies to FH. (d) Detection of IgM antibodies to FI.

Anti-FH and anti-FII antibodies were tested in 9 patients with PT-TMA, 11 renal transplant recipients without PT-TMA, and 10 healthy subjects. IgG reactivity to FH and IgA reactivity to FI were identified in one patient each (nos. 1 and 6, correspondingly [Figures 1(a) and 1(b)]). In both cases, the primary renal disease was complement associated with IgG monoclonality. Significant IgM reactivities to FH and FI were found in several patients with and without PT-TMA as well as healthy controls (Figures 1(c) and 1(d)), with no correlation with disease. Patient 6, who had high IgA anti-FI reactivity, was the only subject in the entire cohort with a negative IgM response to FH and FI.

### 3.7. Renal Biopsies

The major renal biopsy findings are shown in Table 4. Biopsies were performed within 3 weeks after diagnosis of PT-TMA in 10 patients and after more than 1 month in 3 patients, all with late-onset PT-TMA. Light microscopy demonstrated thrombi in the glomeruli in 4 patients, 3 with early PT-TMA, and 1 with late recurrent HUS. Glomerular congestion and fibrinoid necrosis were found in 4 patients, and myxoid changes and mesangiolysis in 3; 4 patients did not have any TMA-specific finding (nos. 6, 9, 10, and 13). The immunofluorescence test was positive in all but one patient. Complement C3 deposits were demonstrated in 7 patients, and C4 deposits in 3 patients. Six patients were positive for immunoglobulins, with IgG being the most common. Comparison of the immunofluorescence results with patients with chronic transplant glomerulopathy yielded no significant difference (data not shown).

Deposits were found in 6 patients (nos. 4, 6, 7, 8, 10, and 11), in the glomerular basement in 3 patients, in the subendothelium in 2, and in the mesangium in 1. Only one of them had a primary immune complex renal disease (Patient 6), and five of them were tested for PEP + IF and were found to be positive. Subendothelial electron lucency was noted in 3 patients, and one patient had fibrin tactoids. Of the 10 patients with full biopsy results, 3 did not have any TMA-specific findings (nos. 6, 10, and 13). In 2 of them, dense deposits were present, and in the third, all findings were related to chronic transplant glomerulopathy, with no apparent PT-TMA involvement of the kidney (Table 4).

Thus, the classical finding of glomerular thrombi was not prominent in our cohort, but findings suggesting a humoral immunological reaction, as manifested by the dense deposits and the positive PEP + IF test, were more remarkable.

### 3.8. PT-TMA Prognosis

The prognosis was generally very poor (Table 3). Within 2 years of presentation with PT-TMA, 6 patients (nos. 6, 8, 9, 11, 12, and 13), including 5 in the late-onset group, had graft failure and required dialysis. Three patients (nos. 3, 7, and 10) showed deterioration in renal function. In the early-onset group, 3 patients (nos. 1, 2, and 5) showed improvement in renal function, and they remained stable under eculizumab treatment.

Three patients were diagnosed with Epstein–Barr virus (EBV)–associated post-transplant lymphoproliferative disorder (EPTLD) 5 years or more after transplantation (nos. 1, 2, and 7), but blood EBV polymerase chain reaction (PCR) was negative in all, and serology was consistent with latent infection. Two of them received eculizumab for 5 years or more, and the third had a severe disease that manifested as relapsing episodes of HUS from the age of 2. Patients 2 and 7 had central nervous system involvement and died of the disease. These patients are described in more detail in the Supporting Information (available here).

Five patients (nos. 2, 3, 4, 7, and 11) died during follow-up at a mean age of 47 years.

## 4. Discussion

Among the cohort of 13 patients with PT-TMA, 9 (69%) had at least one risk factor besides CNI treatment. Patients could be divided into two distinct groups by the time from renal transplantation to PT-TMA onset. Overall, the prognosis was bad, in agreement with the previous studies [1, 2], with rates of 46% for need for dialysis within 2 years and 39% for death (at mean age 47 years). However, some of the patients with early-onset PT-TMA improved with treatment.

Retrospective data analyses and extensive laboratory studies have demonstrated multiple thrombotic effects of CNIs and an association with TMA [15–24]. Although extensive use of these drugs makes renal transplant recipients more prone to the development of TMA than the general population [25], the rate of PT-TMA is still relatively low. Therefore, we assumed that other risk factors may play a role. This study showed that most patients have at least one additional risk factor regardless of the use of CNIs.

The patients with PT-TMA in our study were divided into two groups according to the time of presentation. The early-onset group, in which PT-TMA occurred within 6 months after transplantation, was characterized by a positive DSA test, complement-associated primary renal disease, antibodies directed to complement components, and acute rejection. The late-onset group, in which PT-TMA occurred 3 years or more after transplantation, was characterized by infections. Although these findings are based on small numbers, they suggest that the different stages patients undergo after transplantation expose them to different PT-TMA triggers. Physicians treating transplant recipients should be aware of the risk factors, especially in the first 6 months after transplantation, and maintain a high index of suspicion for PT-TMA when late infections occur.

Infections can induce TMA by direct damage to the endothelium and via complement activation [26, 27]. They are associated with TMA in about 50% of non–transplant recipients [2]. The finding that 3 of our patients (23%) had infection-associated PT-TMA supports the notion that infections may also play a major role in the development of PT-TMA.

Antibodies against FH have been reported in approximately 10% of patients with atypical HUS [3]. Deletion of both copies of CFHR1 has been proposed to favor the development of anti-FH antibodies, although FHR1 deficiency alone is not sufficient to trigger their generation [3, 4, 28]. In our cohort, IgG and IgA antibodies directed at FH and FI, respectively, were detected in 2 of 9 patients tested (22%). In both, immunofixation detected significant IgG clones but no deletions of CFHR1.

Earlier studies have shown that IgM autoantibodies may exert protective activity in several autoimmune diseases [10, 29–31]. In our study, significant IgM reactivity was found in several of the renal transplant recipients with and without PT-TMA as well as in the healthy controls, and it was apparently not associated with disease activity. The finding of IgM reactivity against FH and FI in both healthy subjects and transplant recipients supports the assumption that it is not necessarily pathogenic and may have an immunomodulatory function.

Renal injury in TMA is caused by a vascular pathological process affecting the renal arterioles and glomeruli. Thrombi and myxoid changes can be seen in the arterioles and in the glomeruli, and endothelial swelling leads to narrowing of the glomeruli, glomerular congestion, and mesangiolysis [32, 33]. In our cohort, only 4 patients had the classical TMA finding of the glomerular thrombi; they either had early PT-TMA or their original disease was HUS. In 4 patients, no TMA-specific findings were seen by light microscopy, and 3 of them did not have any TMA-specific finding.

Electron microscopy analysis showed dense deposits in 6 patients. Only one of them was known to originally have original immune complex renal disease, and 5 of them who underwent the PEP + IF test were all positive. Taken together, these findings suggest that in some PT-TMA patients, the humoral immune system plays a prominent pathogenic role in the renal graft.

Renal transplant recipients are at increased risk of EBV-associated EPTLD, usually within the first year after transplantation, with an overall incidence of 0.8%–2.5% [34]. The development of EPTLD is greatly affected by the intensity of the immunosuppression and is usually associated with the use of thymoglobulin, tacrolimus, and mycophenolate mofetil. It is also responsive to immunosuppression reduction. Therefore, many centers monitor quantitative EBV PCR in high-risk patients and reduce immunosuppression when EBV DNA level rises [35]. The pathogenesis of EPTLD involves mainly the decrease in T-cell surveillance that allows the virus to invade B-cells and cause malignant transformation [34]. However, it is possible that defects in other components of the immune system might increase the risk. Alternative complement pathway (ACP)–EBV interactions are thought to regulate immune control of infection, viral evasion potential, and development of EPTLD. Importantly, EBV cellular invasion triggers membrane-associated activation of the ACP [36, 37]. However, EBV can evade immune surveillance by inhibiting the ACP [38].

In our PT-TMA cohort, EPTLD developed in 3 patients with marked complement dysfunction which might have enabled the virus to evade immune system surveillance. Two of them had received long-term eculizumab, and the other had a severe disease that manifested as relapsing episodes of HUS from the age of 2. The finding that EBV was negative in blood but positive in tissues suggests a local rather than a systemic process, indicating that in certain conditions, EBV-PCR surveillance might not be conclusive.

The main limitations of this study are the small number of patients and the collection of data from a single clinical center. Further studies are warranted to validate our findings in larger multicenter cohorts.

In summary, other potential risk factors besides CNI use can probably trigger PT-TMA. More research is required to clearly identify these factors and determine how they trigger PT-TMA and whether they act in concert or independently. Our results also distinguish two groups of PT-TMA by lag time from transplantation, early (within 6 months) and late (after 3 years or more), with different characteristics. The development of EPTLD in 3 of our patients warrants further consideration of the use of immunosuppressants in patients with marked complement dysfunction.

## Figures and Tables

**Table 1:** Clinical and laboratory characteristics of patients with post-transplant thrombotic microangiopathy.

Characteristics	All patients (*N* = 13)	Early PT-TMA (*N* = 6)	Late PT-TMA (*N* = 7)	*p* value (early vs. late)
Age (years)	41.7 ± 11.3	48.8 ± 8.8	35.6 ± 9.7	0.026
Males	5 (39%)	1 (17%)	4 (57%)	0.13
Days from transplant to PT-HUS, median (IQR)	1134 (58; 4768)	58 (10.75; 100)	4454 (2085; 5590)	0.003
C3 (mg/dL) (normal range 90–180 mg/dL)	71 ± 11.6	72.8 ± 15.5	69.1 ± 7.7	0.6
C4 decreased	2/13 (15%)	1/6 (17%)	1/7 (14%)	0.9
Hb (g/dL)	8.8 ± 0.7	9 ± 0.6	8.6 ± 0.8	0.24
Positive schistocytes	9/11 (82%)	3/5 (60%)	6/6 (100%)	0.09
PLT (K/μL)	91 ± 37	103 ± 29	81 ± 43	0.3
LDH (U/L)	824 ± 276	800 ± 357	841 ± 232	0.83
Decreased haptoglobin	8/12 (67%)	3/6 (50%)	5/6 (83%)	0.22
Pretreatment Cr (mg/dL)	2.5 ± 1.4	2.5 ± 1.8	2.5 ± 1.1	0.96

*Note:* Data are expressed as *n* (%) or mean ± SD, unless otherwise stated.

Abbreviations: Cr, creatinine; Hb, hemoglobin; IQR, interquartile range; LDH, lactate dehydrogenase; PLT, platelets; PT-TMA, post-transplant thrombotic microangiopathy.

**Table 2:** Potential risk factors for post-transplant–thrombotic microangiopathy besides CNI treatment.

Pt. no.	Donor-specific antibodies	Acute rejection	Primary renal complement–associated disease	Anti-FH or anti-FI antibodies	Genetics	Infection
*Early-onset PT-TMA*						
1	No	No	Yes	Yes	No	No
2	Yes	Yes⁣^∗^	No	No	No	No
3	No	No	No	No	NT	No
4	Yes	Yes	No	NT	NT	No
5	Yes	No	Yes	No	No	No
6	Yes	No	Yes	Yes	No	No

*Late-onset PT-TMA*						
7	NT	No	Yes	NT	No	No
8	No	No	No	No	No	No
9	NT	No	No	NT	No	No
10	No	No	No	No	No	Yes
11	No	No	No	No	NT	Yes
12	NT	No	No	NT	No	Yes
13	No	No	Unknown	No	No	No

Abbreviations: CNI, calcineurin inhibitor; FH, factor H; FI, factor I; NT, not tested; PT-TMA, post-transplant thrombotic microangiopathy.

⁣^∗^Patient 2 had suspected antibody-mediated rejection.

**Table 3:** Clinical data of patients with post-transplant thrombotic microangiopathy.

Pt. no	Age (yr)/sex	Primary renal disease	Time from KTX to PT-TMA	C3⁣^∗^ (mg/dL)	C4	Hb (g/dL)	Sch	PLT (K/μL)	LDH (U/L)	Hapto	Pre-Tx Cr (mg/dL)	Baseline treatment	PT-TMA treatment	Outcome
*Early-onset PT-TMA*														
1	45/F	Monoclonal- associated DDD	4 days	59	N	9.1	N	55	646	Low	5.3	Tac + MMF	PP, IVIG, thymo, and eculizumab	After 2 years, Cr 1.2RCC and EPTLD diagnosed 7 years after KTX
2	49/F	Thin basement membrane disease	13 days	58	Low	9.7	NA	128	NA⁣^∗∗^	Low	4.2	Tac + MMF	PP, pulse steroids, IVIG, and eculizumab	After 2 years, Cr-1.3 EPTLD diagnosed 6 years after KTX died after 2 years
3	38/F	Renal artery stenosis d/t FMD, and HTN	52 days	87	N	9.3	Pos	121	707	N	1	Tac + MMF	PP, pulse steroids, IVIG, and eculizumab	After 2 years Cr-2.6d died after 4.5 years
4	62/F	FMF	64 days	88	N	8	Pos	80	1434	Low	0.75	Tac + MMF	PP, pulse steroids, IVIG, thymoglobulin, and rituximab	Died after 1.5 years Cr 1.2 at death
5	4/F3	aHUS	92 days	86	N	8.7	N	118	643	N	2	Tac + MMF	PP, pulse steroids, and eculizumab	After 2 years Cr-1.4.

**Pt. no**	**Age/sex**	**Original renal disease**	**Time from transplant PT-TMA**	**C3**⁣^∗^	**C4**	**Hb**	**Sch**	**PLT**	**LDH**	**Hapto**	**Pre-tx Cr (mg/dL)**	**Baseline tx**	**PT-TMA Tx**	**Outcome**

6	56/M	Monoclonal-associated C3 nephropathy	124 days	59	N	9.4	Pos	117	572	N	2	Tac + MMF	PP, pulse steroids, and valcade	Dialysis started after 8 months

*Late-onset PT-TMA*														
7	22/F	aHUS	3.08 years	63	L	8.9	Pos	44	996	NT	0.9	Tac + MMF	PP	After 2 years Cr 2.0. Dialysis started 13 years after KTX and EPTLD was diagnosed. Died after 7 years.
8	24/M	Nephronophthisis	5.7 years	69	N	9.4	Pos	36	508	Low	1.5	Tac + MMF	PP, pulse steroids, and eculizumab	Dialysis started after 2 months
9	39/M	IgA nephropathy	6.04 years	73	N	7.7	Pos	108	1009	Low	3.5	Sirolimus + MMF	Conservative	Dialysis started immediately
10	42/M	Vesicoureteral reflux	12.2 years	80	N	9.4	Pos	118	1000	Low	2.4	Tac + Imuran	PP, pulse steroid, and IVIG	After 2 years Cr 3.5
11	34/M	Unknown	13.9 years	70	N	7.5	Pos	28	875	N	4.1	Tac + MMF	PP, pulse steroids, cytoxan, and eculizumab	Dialysis started immediately. Died after 3 years
12	39/F	Chronic tubulointerstitial disease	15.3	56	N	8.3	Not tested	119	988	Low	2.2	Tac + Imuran	Conservative	Dialysis started after 21 months
13	49/F	Unknown	18.3 years	73	N	8.7	Pos	115	508	Low	3	Tac + MMF	Conservative	Dialysis started after 9 months

Abbreviations: aHUS, atypical hemolytic uremic syndrome; Cr, creatinine; DDD, dense deposit disease; EPTLD, EBV-associated pretransplant lymphoproliferative disorder; FMD, fibromuscular dysplasia; Hapto, haptoglobin; Hb, hemoglobin; HTN, hypertension; IVIG, intravenous immunoglobulin; KTX, kidney transplant; LDH, lactate dehydrogenase; MMF, mycophenolate mofetil; mo, months; N, normal; NA, not available; PLT, platelets; Pos, positive; PP, plasmapheresis; PT-TMA, post-transplant thrombotic microangiopathy; RCC, renal cell cancer; Sch, schistocytes; TAC, tacrolimus; Thymo, thymoglobulin; Tx, treatment; yr, years.

^∗^C3 normal range: 90–180 mg/dL.

^∗∗^According to medical records, LDH was elevated but we could not obtain the precise value.

**Table 4:** Infections, DSA, PEP + IF, renal biopsy, and genetic results of patients with PT-TMA post-transplant–thrombotic microangiopathy.

Pt. no.	DSA	Infection-associated PT-TMA	PEP + IF	Renal biopsy						Genetic analysis
				IF	LM⁣^∗^	EM⁣^∗^	Final diagnosis	Banff 2022	Time from PT-TMA to renal biopsy	
*Early-onset PT-TMA*										
1	N	No	IgG lambda	C3	Glomerular congestion and fibrinoid necrosis	Subendothelial lucency	Recurrence of original disease (DDD)	⁣^∗∗^	2 days	No pathogenic variants in complement genes
2	P	No	IgG kappa biclonal	MAC	Glomerular thrombi, glomerular congestion, mesangiolysis, glomerulitis, and capillaritis	Not reported	C4d-negative antibody-mediated rejection	Probably AMR	3 days	No pathogenic variants in complement genes
3	N	No	IgG kappa	C3	Fibrinoid necrosis, glomerular thrombi, and mesangiolysis	Subendothelial lucency	TMA	⁣^∗∗^	21 days	NT
4	P	No	IgG kappa, IgG lambda, and IgA lambda	C3, C4d	Glomerular congestion and myxoid changes	GBM deposits	HUS and antibody-mediated rejection	Active AMR	0 days	NT
5	P	No	IgG kappa	IgG	Glomerular thrombi and mesangiolysis	Fibrin tactoids, subendothelial lucency	TMA	⁣^∗∗^	2 days	Del (CFHR3-CFHR1)/normal allele
6	P	No	IgG kappa	C3	No HUS-specific findings	Mesangial deposits	Recurrence of original disease (C3 nephropathy)	⁣^∗∗^	0 days	No pathogenic variants in complement genes

*Late-onset PT-TMA*										
7	Not tested	No	NT	IgG, C3, C4, and C1q	Glomerular thrombi, fibrinoid necrosis, and glomerular congestion	GBM deposits	HUS + CTG	⁣^∗∗^	8 days	No pathogenic variants in complement genes
8	N	No	Lambda	Negative	No result	Subendothelial deposits	Tissue insufficient for diagnosis	Not diagnostic	21 days	Del (CFHR3-CFHR1)/normal allele
9	Not tested	No	No peak	IgM	No HUS-specific findings	Not reported	CTG	⁣^∗∗^	145 days	Del (CFHR3-CFHR1)/normal allele
10	N	Yes, hospitalization d/t viral infection	IgM kappa	IgG, C3, and C4d	No HUS-specific findings	GBM deposits	CTG	Chronic active antibody–mediated rejection	1 day	Del (CFHR3-CFHR1)/normal allele
11	N	Yes, buccal cellulitis d/t infection of teeth	IgG lambda biclonal	IgG, C4, IgM, Kappa	Fibrinoid necrosis and myxoid changes	Subendothelial deposits	Focal and segmental necrotizing and exudative glomerulonephritis with cellular crescents	⁣^∗∗^	36 days	NT
12	Not tested	Yes, viral meningitis	No peak	C3, C4d	Myxoid changes	No HUS-specific findings or deposits	CTG	Chronic active antibody–mediated rejection	127 days	Del (CFHR3-CFHR1)/del (CFHR3-CFHR1) and CD 46 haplotype risk for C3GN
13	N	No	No peak	IgG	No HUS-specific findings	No HUS-specific findings or deposits	CTG	⁣^∗∗^	14 days	No pathogenic variants in complement genes

Abbreviations: CTG, chronic transplant glomerulopathy; DSA, donor-specific antibodies; EM, electron microscopy; GBM, glomerular basement membrane; HUS, hemolytic uremic syndrome; IF, immunofluorescence; LM, light microscopy; MAC, membrane attack complex (C5b-9); N, negative; NT, not tested; P, positive; PEP + IF, protein electrophoresis and immunofixation; PT-TMA, post-transplant thrombotic microangiopathy.

⁣^∗^Only TMA-specific findings are shown.

⁣^∗∗^No chronic or acute rejection.

## Data Availability

The data that support the findings of this study are available from the corresponding author upon reasonable request.

## Supporting Information

This section contains the results of the immunofixation tests and the clinical description of patients with PT-TMA and EPTLD.
