# Supplementary material for: Comprehensive Analysis of Thrombotic Microangiopathy Following Renal Transplantation
Source: Int J Nephrol. 2024 Dec 24;2024:4396051. doi: 10.1155/ijne/4396051 (PMC11688143; doi:10.1155/ijne/4396051)
Supplement: Supporting Information — Additional supporting information can be found online in the Supporting Information section. [file 4396051.f1.docx]

# Supplementary Material

**Immunofixation Tests**

Immunofixation test results were available for 12 of the patients, and 9 of them were found positive (Table 4). The 2 bands of patients #1 and 6, whose original disease was monoclonal associated DDD or C3 glomerulonephritis, respectively, were strong and could be quantified. In contrast, the other 7 clones were weaker, smeared, and could not be quantified. IgG kappa was the most common (6 patients), but IgM, IgA and lambda were also found. In Patient #4, 2 different results were observed; first, IgG kappa, and then, both IgA lambda and IgG lambda. We did not find a significant correlation between the band in the blood and the IF results of the renal biopsy, and except for Patient #1, the bands also did not correlate with the reactivities to FH and FI. Thus, it seems that these bands do not cause direct damage to the kidney or influence FH or FI. Bone marrow biopsies were performed in 5 patients and were all found normal.

**Clinical Description of Patients with PT-TMA and EBV-Associated PTLD**

**Patient 1**

Patient 1 had IgG lambda monoclonal-associated dense deposit disease and tested positive for anti-FH antibodies. The paraprotein measured 2 gr/dL, and bone marrow biopsy was normal. At age 30 years, she gave birth to a healthy girl, but after 6 days, the infant was hospitalized for acute kidney injury. Laboratory results showed elevated creatinine level, hematuria, proteinuria, and leukocyturia, and immunofixation test showed IgG lambda paraprotein. The patient underwent a double-volume exchange transfusion and recovered immediately. It was speculated that the patient had transferred the paraprotein that inhibited FH to her daughter, causing the disease.^1^

The patient’s renal function deteriorated, and at age 32 years she started dialysis. Two years later, she received a kidney from a living donor. However, the disease recurred in the transplant, and she was treated with rituximab and plasmapheresis. At age 42 years, she resumed dialysis, and 3 years later received another kidney from a non-living donor. Four days after transplantation, levels of C3, hemoglobin, platelets, and haptoglobin decreased, LDH and creatinine increased, and DSA test was negative. Renal biopsy showed minimal glomerular basement membrane thickening with scattered polymorphonuclear cells within the capillary lumen; C3 was +1 mesangial, C4d was negative, and subendothelial electron lucency was detected by electron microscopy. PT-TMA was diagnosed, and treatment with plasmapheresis, intravenous immunoglobulin, thymoglobulin and eculizumab was started. Creatinine level improved from 5.3 to 1.2 mg\dL.

Thereafter, the patient was treated with cyclosporine and mycophenolate mofetil 360 mgX3/d, prednisone 5 mgX1/d in combination with eculizumab 900 mg every 2 weeks for 7 years.

At age 52 years, she presented with fever of unknown origin. Renal cell carcinoma was diagnosed and excised, but the fever continued. Positron emission tomography-computed tomography scan showed enlarged lymph nodes above and under the diaphragm suggestive of lymphoma. Lymph node biopsy revealed EPTLD. PCR test showed T-cell monoclonality, making it unlikely that the disease developed from the abnormal IgG lambda clone. Blood EBV PCR was negative, and EBV serology was consistent with latent infection.

**Patient 2**

Patient 2 had basement membrane disease that deteriorated during pregnancy. At age 27 years, she started dialysis, and 6 years later, she received a transplant from a living hepatitis C virus (HCV)-positive donor. The graft failed after 1.5 years, and the patient resumed dialysis for another 15 years. During that time, she was treated successfully for HCV. At age 49 years, she received a second transplant from a living donor. At 13 days after transplantation, levels of hemoglobin, haptoglobin, platelets, and C3 and C4 were decreased, DSA increased to 7000 MFI, and creatinine increased from 0.9 to 4.2 mg/dL. Renal biopsy showed micro-thrombi, mesangiolysis, capillary congestion, glomerulitis~~,~~ and peritubular capillaritis. Immunofluorescence was negative for immunoglobulins, C3, and C4d but positive for complement membrane attack complex (C5b-9).

The patient was diagnosed with PT-TMA associated with C4d negative antibody-mediated rejection and started treatment with pulse steroids, plasmapheresis, IVIG, and eculizumab. Creatinine decreased to 1.3 mg/dL. Two years later, she tested positive for biclonal IgG kappa. The clones were small and could not be quantitated. Bone marrow biopsy was not performed.

After the second transplant, the patient received tacrolimus, mycophenolate mofetil 500 mgX2/d, and prednisone 5mgX1/d in combination with eculizumab 900 mg every 2 weeks for 5 years, at which point she presented with convulsions. Magnetic resonance imaging showed lesions in the left occipital lobe and right fronto-temporal and fronto-parietal areas. EPTLD was diagnosed by brain biopsy. Blood EBV PCR was negative, and EBV serology corresponded to latent infection. Immunosuppression and eculizumab were stopped. Rituximab was administered, and the disease regressed, but after 1.5 years, it relapsed. Despite treatment with rituximab, CCNU, procarbazine, and nivolumab, the patient died 6 months later.

**Patient 7**

Patient 7 presented with HUS at age 2 years and needed 3 dialysis treatments until her kidneys recovered. Thereafter, she was lost to nephrological follow-up until age 18 years, when she presented with weight loss and weakness. Laboratory results revealed advanced renal failure, and at age 19 years, she received a preemptive renal transplant from her brother. Creatinine level was 0.9 mg\dL for 4 years, when the disease relapsed. Renal biopsy showed microthrombi and glomerular basement membrane thickening. The patient was treated with plasmapheresis and dialysis was administered for 3 months, until the patient recovered and creatinine stabilized at 2 mg/dL. CNI treatment was stopped and replaced with mycophenolate mofetil 500 mgX2/d and prednisone 7.5 mgX1/d. Four years later, at age 27 years, when the patient was 12 weeks pregnant, the disease relapsed and creatinine increased to 4.7 mg/dL. Plasmapheresis was administered and the pregnancy was terminated. Creatinine level decreased to 2.5 mg/dL.

Thereafter, renal function gradually deteriorated, and after 5 years, at age 31, the patient started hemodialysis. Four months later she presented with a 2-week history of recurrent episodes of amnesia, involuntary right eye movements, and right arm paresthesia. Brain magnetic resonance imaging showed two frontal lesions in the left hemisphere, and brain biopsy revealed EBV-positive diffuse large B cell lymphoma. Blood EBV PCR was negative, and EBV serology was consistent with latent infection. Treatment consisted of rituximab, whole brain radiation and lenalidomide. The patient died after 7 years.

**References**

1. Dolfin T, Pomeranz A, Korzets Z, et al. Acute renal failure in a neonate caused by the transplacental transfer of a nephrotoxic paraprotein: successful resolution by exchange transfusion. *Am J Kidney Dis.* 1999;34:1129–1131.
